# Increased femoral cartilage thickness in acne patients using isotretinoin: could it be a sign of early osteoarthritis?

**DOI:** 10.1007/s00403-024-03264-x

**Published:** 2024-08-12

**Authors:** Sevgi Kulaklı, Asude Cevher Elmas Telli, İlker Fatih Sarı, Işıl Deniz Oğuz, Fazıl Kulaklı

**Affiliations:** 1 Department of Dermatology and Venereology, Giresun University Faculty of Medicine, Giresun, 28200 Turkey; 2 Department of Physical Medicine and Rehabilitation, Giresun University Faculty of Medicine, Giresun, 28200 Turkey

**Keywords:** Acne, Isotretinoin, Femoral cartilage thickness, Osteoarthritis, Ultrasound

## Abstract

Vitamin A derivatives have inhibitory effects on cartilage tissue, such as decreasing chondrocyte proliferation and collagen synthesis, and increasing the loss of glycosaminoglycans and proteoglycans. Therefore, isotretinoin (a vitamin A derivative) may play a role in the pathogenesis of cartilage-related diseases like osteoarthritis by affecting the balance of cartilage tissue. The aim of this study was to evaluate the distal femoral cartilage thickness in acne patients under the systemic isotretinoin therapy and to determine whether it constitutes a risk factor for the development of osteoarthritis. The study included 52 patients (42 female, 10 male, mean age 23.31 ± 3.89 years) who were prescribed systemic isotretinoin for acne and completed at least 3 months of treatment, along with 45 healthy controls ((35 female, 10 male, mean age 23.85 ± 4.77 years). Bilateral distal femoral cartilage thickness was measured by ultrasonography before isotretinoin treatment and after the completion of the third month of treatment. After treatment, a statistically significant increase was found in the thickness of the right medial, right lateral, left medial, left lateral, and left intercondylar cartilage (*p* = 0.014, 0.012, 0.019, 0.027, 0.002, respectively). There was also an increase in the right intercondylar cartilage thickness, but this was not statistically significant (*p* = 0.1). Systemic isotretinoin seems to make cartilage thicker. The increase in femoral cartilage thickness observed after short-term isotretinoin treatment might be an indicator of very early-stage osteoarthritis. Extended follow-up studies with larger participant pools are necessary to substantiate this result.

## Introduction

Acne vulgaris is a chronic inflammatory disease of the pilosebaceous unit. It has a multifactorial etiology and affects approximately 85% of adolescents and young adults [1]. Isotretinoin (13-cis-retinoic acid), a derivative of vitamin A, is the most effective agent used in the treatment of acne. However, it can lead to various side effects in multiple organ systems, including the skin, mucosa, reproductive system, liver, eyes, and musculoskeletal system [2]. The main musculoskeletal side effects associated with isotretinoin include myalgia, arthralgia, sacroiliitis, spondyloarthropathy-like symptoms, hyperostosis, extraspinal calcification, enthesitis, arthritis, costochondritis, osteoporosis, and premature epiphyseal closure [3–6].

It is known that vitamin A derivatives play an important role in cartilage and skeletal development by inhibiting the chondrogenic differentiation of mesenchymal cells and chondrocyte differentiation [7]. Various in vitro studies have shown that retinoic acid derivatives increase the number of hypertrophic chondrocytes, enhance the loss of glycosaminoglycans and proteoglycans in articular cartilage, reduce type 2 collagen synthesis, decrease the adhesion of chondrocytes to the extracellular matrix, and increase the activity of aggrecanase, matrix metalloproteinase (MMP) 13, and transglutaminase [8–15]. Due to these effects of retinoic acids, it has been suggested in various studies that they may play a role in the pathogenesis of osteoarthritis (OA). One of these studies found that retinoic acid levels were elevated in the synovial fluids of donors with OA compared to controls [7].

In this study, we aimed to evaluate the effect of isotretinoin treatment on femoral cartilage thickness (FCT) in patients with acne and, based on this, to determine if it poses a risk factor for the development of OA.

## Methods

This follow-up study included patients aged ≥ 18 years who were started on systemic isotretinoin treatment for acne. The patients’ age, sex, occupation, body mass index (BMI), smoking status, and exercise habits were recorded. Those who exercised for at least 30 min per day or at least 3 days per week at moderate to high intensity were considered to be actively exercising. Patients were instructed not to make any changes to their daily routines (such as diet, exercise, or smoking) during the study period. Before starting the treatment, the severity of acne was determined by a Dermatology specialist using the Global Acne Grading System (GAGS). According to this grading system, acne severity was recorded as mild, moderate, severe, or very severe. In GAGS, the global score is obtained by multiplying the anatomical location factor (forehead: 2, right cheek: 2, left cheek: 2, chin: 1, nose: 1, chest and upper back: 3) by the severity factor of the lesions (none: 0, ≥ 1 comedone: 1, ≥ 1 papule: 2, ≥ 1 pustule: 3, ≥ 1 nodule: 4) and summing the local scores. A global score ranging from 1 to 18 indicates mild, 19–30 moderate, 31–38 severe, and > 39 indicate very severe acne [16].

The control group was comprised of healthy adults who were matched in terms of age and gender. They were non-pregnant individuals without endocrine, renal, hepatic, or rheumatic diseases, malignancy, or any history of bone metabolism-affecting medication use, knee trauma, or surgery.

**Fig. 1:**
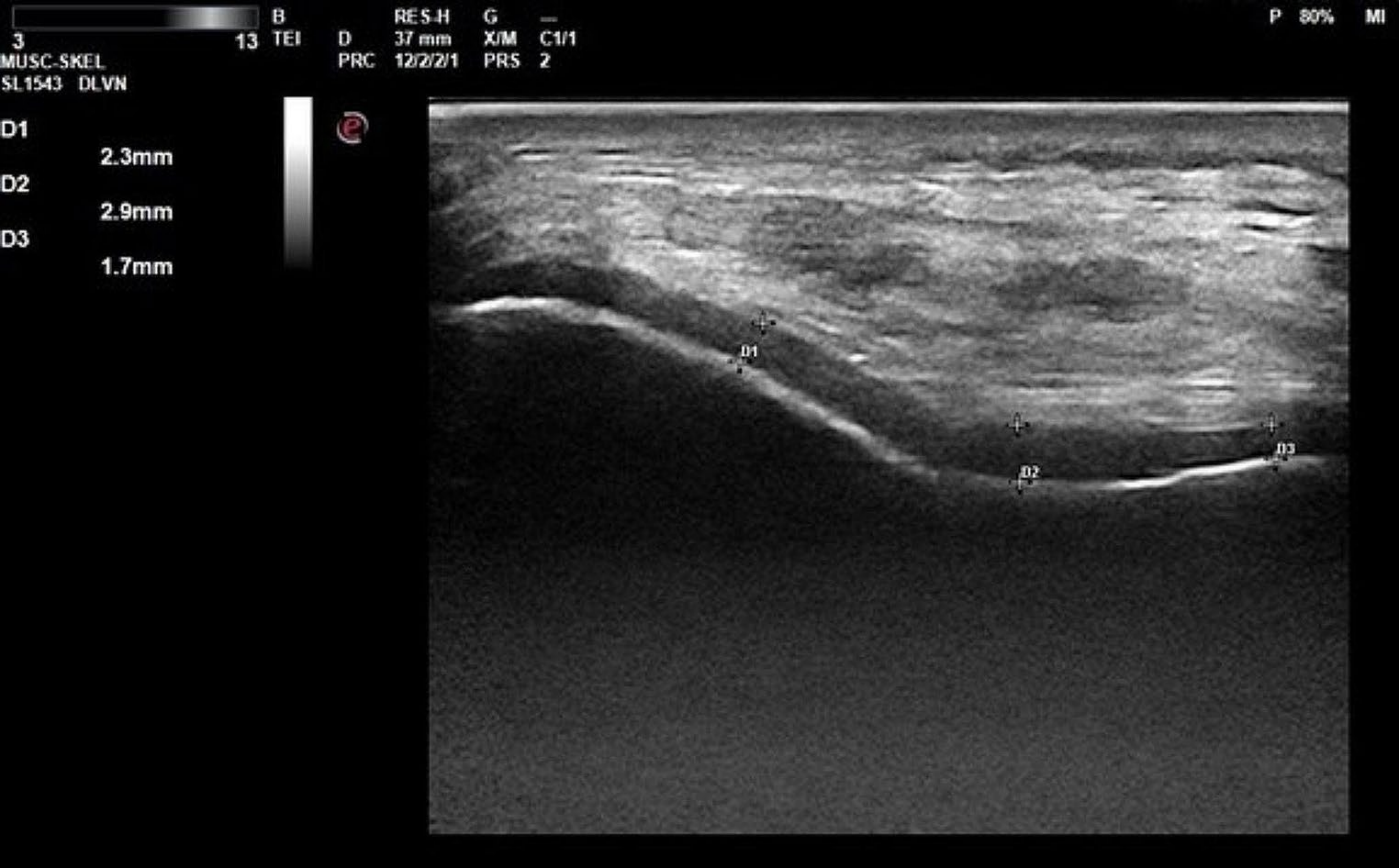
Ultrasonografic measurement of right distal femoral cartilage thickness. D1, right lateral condyle; D2, right intercondylar area; D3, right medial condyle

All participants were evaluated by a Physical Medicine and Rehabilitation physician for knee pain and morning stiffness, and a knee examination was conducted (checking for crepitus and knee joint enlargement). The distal FCT was measured via ultrasonography by a different Physical Medicine and Rehabilitation physician who was blinded of the subjects’ clinical and medication status. The ultrasonographic examination was conducted using a 1–22 MHz linear ultrasound probe (MyLabSix, Esoata Group, Genoa, Italy) with the patient in a supine position and their knees in maximum flexion. The probe was placed in the axial position at the suprapatellar region. Measurements were taken at the medial condyle, lateral condyle, and mid-point of the intercondylar area in both knees (Fig. 1).

The patients were evaluated once more after completing the third month of treatment. The severity of acne during the follow-up was recorded by the same dermatology specialists, along with the total isotretinoin dose and the duration of treatment. Subsequently, the Physical Medicine and Rehabilitation physicians conducted a knee examination and performed the same ultrasonographic measurements.

### Statistical analysis

The data were analyzed using IBM SPSS software (version 23.0; SPSS, Chicago, IL, USA). Numerical data were presented as mean ± standard deviation or median, depending on the distribution of the descriptive data. Categorical data were stated as frequencies and percentages. To compare numerical data between the two groups, the Independent Samples t-test was used if the normality assumption was met, and the Mann-Whitney U test was used if it was not. The chi-square or Fisher’s exact tests were used to compare categorical data between the two groups. The Paired Student’s t-test was used to compare the means, while the Wilcoxon signed ranks test was used to compare the medians of two related groups. The Spearman correlation test was used to evaluate the correlation between treatment duration and cumulative isotretinoin dose with changes in FCT. A p value of < 0.05 was considered statistically significant.

## Results

The study included 52 acne patients (42 female, 10 male) with a mean age of 23.31 ± 3.89 years, and 45 healthy controls (35 female, 10 male) with a mean age of 23.85 ± 4.77 years. There were no significant differences between the patient and control groups regarding age, gender, BMI, occupation, smoking status, and exercise habits. No significant differences were found in FCT between the two groups. The demographic characteristics and FCT of the patient and control are presented in Table 1. Before treatment, none of the patients or controls had knee pain or morning stiffness, and no crepitus or knee joint enlargement was detected in the knee examinations.

**Table 1:** Demographic characteristics and femoral cartilage thickness of the patients and control

	Patients (*n* = 52)	Control (*n* = 45)	*p*
Age, years, mean ± SD	23.31 ± 3.89	23.85 ± 4.77	0.598†
Gender, n (%) Female Male	42 (80.8) 10 (19.2)	35 (77.8) 10 (22.2)	0.716¶
Occupation, n (%) Student Housewife White collar	31 (59.6) 7 (13.5) 14 (26.9)	21 (46.7) 11 (24.4) 13 (28.9)	0.308¶
Smoking, n (%) Smoker Never smoked	10 (19.2) 42 (80.8)	7 (15.6) 38 (84.4)	0.635¶
Exercise status, n (%) Active Inactive	7 (13.5) 45 (86.5)	2 (4.4) 43 (95.6)	0.17§
BMI (kg/m^2^), mean ± SD	22.54 ± 3.7	23.58 ± 2.93	0.073‡
RMCT (mm), mean ± SD	2.07 ± 0.43	2.09 ± 0.38	0.87‡
RLCT (mm), mean ± SD	2.02 ± 0.34	2.15 ± 0.31	0.055‡
RICT (mm), mean ± SD	2.26 ± 0.51	2.24 ± 0.44	0.518†
LMCT (mm), mean ± SD	2.08 ± 0.35	2.16 ± 0.42	0.694†
LLCT (mm), mean ± SD	2.1 ± 0.44	2.13 ± 0.35	0.795‡
LICT (mm), mean ± SD	2.24 ± 0.49	2.31 ± 0.45	0.465‡

BMI, Body Mass Index; RMCT, right medial condyle thickness; RLCT, right lateral condyle thickness; RICT, right intercondylar thickness; LMCT, left medial condyle thickness; LLCT, left lateral condyle thickness; LICT, left intercondylar thickness; SD, standard deviation. †Mann Whitney U test, ¶Pearson ki kare, §Fisher exact test, ‡Student T test. *p* < 0.05 is statistically significant

Thirty-seven patients (71.2%) were re-evaluated clinically and ultrasonographically at 3 months after starting treatment, eight patients (15.4%) at 4 months, and seven patients (13.5%) at 5 months. The average cumulative dose of isotretinoin received by the patients up to the control time was 2944.23 ± 1003.21 mg. Among the patients, three (5.8%) had mild acne, 23 (44.2%) had moderate acne, 21 (40.4%) had severe acne, and five (9.6%) had very severe acne. The pre-treatment GAGS score of the patients was 29.02 ± 6.76, and it was 7.54 ± 4.11 after treatment. There was a statistically significant reduction in the GAGS score after treatment (*p* < 0.001) (Table 2).

**Table 2:** Pre-treatment and post-treatment global acne grading system scores and femoral cartilage thickness

	Pre-treatment	Post-treatment	*P*
GAGS score, mean ± SD	29.02 ± 6.76	7.54 ± 4.11	**< 0.001¥**
RMCT (mm), mean ± SD	2.07 ± 0.43	2.19 ± 0.4	**0.014#**
RLCT (mm), mean ± SD	2.02 ± 0.34	2.14 ± 0.39	**0.012#**
RICT (mm), mean ± SD	2.26 ± 0.51	2.37 ± 0.48	0.1#
LMCT (mm), mean ± SD	2.08 ± 0.35	2.18 ± 0.39	**0.019#**
LLCT (mm), mean ± SD	2.1 ± 0.44	2.24 ± 0.45	**0.027#**
LICT (mm), mean ± SD	2.24 ± 0.49	2.43 ± 0.52	**0.002#**

GAGS, Global Acne Grading System RMCT; right medial condyle thickness; RLCT, right lateral condyle thickness; RICT, right intercondylar thickness; LMCT, left medial condyle thickness; LLCT, left lateral condyle thickness; LICT, left intercondylar thickness; SD, standard deviation. #Paired samples T test, ¥ Wilcoxon signed ranks test. Bold values, *p* < 0.05

After treatment, one patient (1.9%) reported bilateral knee pain. This patient did not have morning stiffness. Physical examination revealed no crepitus or knee joint enlargement. An increase in medial cartilage thickness in both the right and left knees was observed. However, direct knee radiographs did not show any signs of OA. During follow-up, the patient also developed back pain, and sacroiliitis was detected on sacroiliac magnetic resonance imaging (MRI).

After treatment, a statistically significant increase was found in the thickness of the right medial, right lateral, left medial, left lateral, and left intercondylar cartilage (*p* = 0.014, 0.012, 0.019, 0.027, 0.002, respectively). There was also an increase in the right intercondylar cartilage thickness, but this was not statistically significant (*p* = 0.1). The pre-treatment and post-treatment FCT values of the patients are shown in Table 2.

No significant correlation was found between the change in GAGS score, the duration of treatment, and the cumulative dose of isotretinoin with the changes in FCT (Table 3).

**Table 3:** Correlation between change in femoral cartilage thickness and change in global acne grading system score, treatment duration, and cumulative isotretinoin dose

	Change in GAGS score	Treatment duration	Cumulative dose
Change in RMCT r p	-0.065 0.646	0.165 0.243	0.122 0.388
Change in RLCT r p	-0.194 0.169	0.173 0.22	0.064 0.655
Change in RICT r p	0.02 0.89	0.186 0.187	0.088 0.537
Change in LMCT r p	-0.118 0.403	0.038 0.787	-0.053 0.711
Change in LLCT r p	-0.124 0.38	0.164 0.245	0.041 0.77
Change in LICT r p	-0.061 0.669	0.231 0.1	0.064 0.654

Spearman’s Rho test was used for correlation analysis. GAGS, Global Acne Grading System RMCT; right medial condyle thickness; RLCT, right lateral condyle thickness; RICT, right intercondylar thickness; LMCT, left medial condyle thickness; LLCT, left lateral condyle thickness; LICT, left intercondylar thickness. *P* < 0.05 is statistically significant

## Discussion

Vitamin A derivatives, which are essential for significant physiological and metabolic functions like cell differentiation, gene transcription, morphogenesis, and the immune system, also play a crucial role in chondrogenic development and differentiation [7, 17]. Various studies have reported that retinoic acid derivatives have inhibitory effects on cartilage tissue, inhibiting chondrocyte proliferation and collagen synthesis, increasing the loss of glycosaminoglycans and proteoglycans in joint cartilage, reducing chondrocyte adhesion to the extracellular matrix, and increasing the activity of aggrecanase, MMP13, and transglutaminase [8–15, 18–20]. OA is a common degenerative musculoskeletal disease which consists of cartilage degeneration, narrowing of joint space, formation of osteophytes, subchondral bone changes, and inflammation of the synovial membrane. In OA, the balance between the synthesis and degradation of the components that make up the extracellular matrix in joint cartilage is disturbed, leading to increased degradation [21]. The levels of proteases, particularly MMP13, increase, while glycosaminoglycan levels and collagen synthesis decrease, and the proportion of hypertrophic chondrocytes rises [22]. A study found that retinoic acid levels were elevated in the synovial fluids of donors with OA compared to healthy controls [7]. Based on this information, we hypothesized that isotretinoin, a derivative of vitamin A, might have a role in the progress of OA by causing changes in joint cartilage, and we planned this study accordingly. We used ultrasonographic imaging in our study because it is an inexpensive, easy-to-apply, and suitable method for dynamic imaging, as well as being effective in evaluating and measuring the thickness of distal femoral cartilage [23–25]. Since OA most commonly affects the knee joint, and the measurement of FCT is considered an important tool for the diagnosis and follow-up of OA, we evaluated FCT using ultrasound [21, 26]. In our study, we found a significant increase in distal FCT in acne patients following isotretinoin treatment.

Isotretinoin is a highly effective and frequently used agent in the treatment of acne. However, it can cause various side effects in different organ systems, including the musculoskeletal system [2]. The most common musculoskeletal side effects are myalgia and arthralgia, occurring in approximately 20% of cases [3]. In addition, sacroiliitis, spondyloarthropathy, hyperostosis, extraspinal calcification, enthesitis, arthritis, costochondritis, osteoporosis, and premature epiphyseal closure are rare musculoskeletal side effects associated with isotretinoin [4–6]. Isotretinoin exerts its effects by isomerizing to all-trans retinoic acid (ATRA) in the body [2]. ATRA has been found to increase MMP13 and aggrecanase activity in human cartilage tissue, affect the expression of genes regulating collagen synthesis, and cause hypertrophy in chondrocytes. Due to these effects, it has been suggested that ATRA could play a role in the pathogenesis of OA [7]. There are no studies in the English literature evaluating the relationship between isotretinoin and OA. However, there is one study similar to ours that evaluated the effect of isotretinoin on cartilage thickness. In this study, distal FCT and the thickness of the Achilles and supraspinatus tendons were evaluated before and at the end of the third month of treatment in 15 patients using isotretinoin for acne. Unlike our study, this study reported a decrease in all FCT values post-treatment, but this decrease reached statistical significance only in the lateral condyle area. The authors suggested that isotretinoin treatment led to cartilage thinning by affecting the proteoglycan and water content of the cartilage. In comparison to our study, this previous research had a significantly smaller sample size and did not include a control group. Furthermore, the severity of acne was not specified, nor were any lifestyle factors such as occupation, exercise habits, or smoking status, which could influence cartilage thickness, provided. Additionally, there were no data on pre- and post-treatment cartilage examination findings or patient symptom assessments. During statistical analysis, the distal FCT values were evaluated collectively rather than separately for the right and left sides [27].

Osteoarthritis is a musculoskeletal disease that affects all the structures comprising the joint, especially cartilage, and most frequently involves the knee joint. Major factors involved in the onset and progression of the disease include advanced age, female gender, obesity, trauma, and genetic factors [21]. In the process of OA, the first change occurring in the cartilage matrix is the formation of soft and swollen cartilage due to increased water content. This condition results from the inability of the loosened collagen network to resist the osmotic pressure created by aggrecans. Hence, in the very early stages of OA, an increase in cartilage thickness is detected, while in fully developed and advanced OA, erosion and thinning or loss of joint cartilage occur [27–29]. Cartilage thickness measurement is an important method used in both the initial stage and the determination of the progression of OA. Previous studies have reported that the FCT measured by ultrasound correlates with the cartilage thickness evaluated macroscopically and with histological measurement results [30–32]. In studies evaluating experimental knee OA created in rabbits, Calve et al. found swelling in the knee cartilage in the early stage of OA using MRI [33, 34]. In a study evaluating FCT with ultrasound in patients with pes planus, an increase was detected in the patient group. This increase was suggested to be due to cartilage edema appearing in the early stages of OA [24]. Similarly, in studies conducted on patients with polycystic ovary syndrome, increased FCT was found [25, 35]. In our recent study evaluating FCT in patients with seborrheic dermatitis, we similarly found that cartilage thickness increased seborrheic dermatitis patients [36]. In these studies, similar to ours, the study population predominantly consisted of young adults, and the increase in FCT was attributed to tissue deterioration occurring in the very early phases of OA [24, 25, 35, 36].

The limitations of this study comprise the brief duration of follow-up, the lack of direct radiographic or MRI assessment for evaluating OA, the omission of assessing joints beyond the knee, and the absence of examination regarding the intra- or inter-observer reliability of ultrasound findings.

In conclusion, we believe that the increase in FCT observed after short-term isotretinoin treatment might be an indication of very early-stage OA. Our study population consists of young patients, a demographic where OA is rarely seen. Isotretinoin may be initiating the first hit in the development of OA in these patients. To support this hypothesis, more extensive studies with longer follow-up periods are needed, where patients using isotretinoin are re-evaluated at older ages.

## Data Availability

The datasets generated during and/or analyzed during the study are available upon reasonable request.
